# Supplementary material for: The interactome of intact mitochondria by cross-linking mass spectrometry provides evidence for coexisting respiratory supercomplexes
Source: Mol Cell Proteomics. 2017 Dec 8;17(2):216–32. doi: 10.1074/mcp.RA117.000470 (PMC5795388; doi:10.1074/mcp.RA117.000470)
Supplement: Supplemental Data [file supp_RA117.000470_133922_0_supp_23978_fzffwf.zip › spectra_annotation/mito_spectra_annotation/414-1-8-1-11-1.pdf]

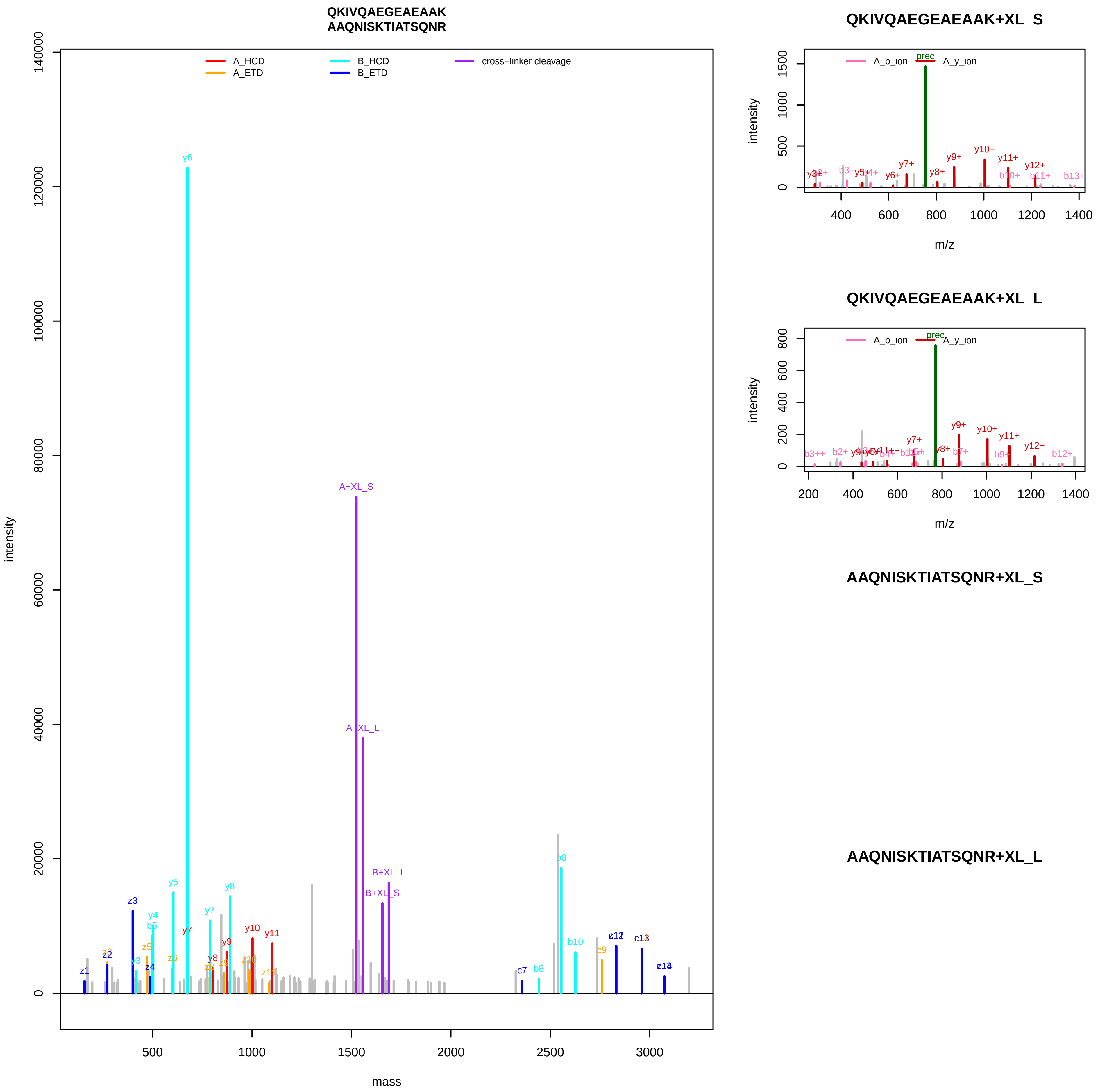
QKIVQAEGEAEAAK
AAQNISKTIATSQNR
A_HCD
A_ETD
B_HCD
B_ETD
cross−linker cleavage
intensity
mass
y6
A+XL_S
A+XL_L
B+XL_L
B+XL_S
b9
b10
c13
c9
c7
b8
y5
y8
y7
y4
z3
y10
y11
y9
y8
y7
z1
z2
z4
z5
z6
QKIVQAEGEAEAAK+XL_S
A_b_ion
A_y_ion
prec
y9+
y10+
y11+
y12+
y7+
y6+
y8+
b13+
m/z
QKIVQAEGEAEAAK+XL_L
y12+
b12+
AAQNISKTIATSQNR+XL_S
AAQNISKTIATSQNR+XL_L
